# Anaphylaxis to Patent Blue V Dye With Clinical Deterioration Following Administration of Suxamethonium and Sensitisation on Skin Testing to Methylene Blue, Patent Blue V Dye and Suxamethonium

**DOI:** 10.7759/cureus.63935

**Published:** 2024-07-05

**Authors:** Edward A Benison, Tomaz Garcez, Lucy Chambers, Andrew Parkes

**Affiliations:** 1 Anaesthesia, Santa Chiara Hospital, Trento, ITA; 2 Immunology, Manchester University NHS Foundation Trust, Manchester, GBR; 3 Anaesthesia, Liverpool University Hospitals NHS Foundation Trust, Liverpool, GBR; 4 Anaesthesia, Manchester University NHS Foundation Trust, Manchester, GBR

**Keywords:** anaesthesia, dye, perioperative, anaphylaxis, drug interactions, allergy

## Abstract

Neuromuscular blocking drugs (NMBAs) and Patent Blue V dye sodium salt 2.5% (Guerbet, Roissy, France) are frequently implicated in perioperative allergic immunoglobulin E (IgE) mediated anaphylaxis. Most cases of anaphylaxis during surgery occur at induction of anaesthesia, although reactions to vital dyes injected into soft tissues often show a delayed onset. We present the case of a female in her 60s who suffered perioperative anaphylaxis to Patent Blue V dye and possibly suxamethonium during oncological breast surgery. Allergy clinic follow-up confirmed sensitivity to both drugs which may explain the unusual bi-phasic nature of the reaction. Intradermal testing also found cross-sensitivity to methylene blue, but not to other common allergens or NMBAs. This case demonstrates the importance of thorough post-anaphylaxis follow-up and raises the possibility of cross-sensitivity between unrelated compounds.

## Introduction

Anaphylaxis is defined as 'a severe life-threatening generalised or systemic hypersensitivity reaction' [1] which is predominantly, but not universally, allergic in aetiology. Allergic anaphylaxis is usually immunoglobulin E (IgE) mediated and presents with life-threatening features [2]. Perioperative hypersensitivity reactions are graded from 1 to 5 based on their severity, with the term 'anaphylaxis' encompassing grades 3 to 5. Grade 3 anaphylaxis typically presents with a cardiovascular compromise with or without associated mucocutaneous and respiratory features. The 6th UK National Audit Project (NAP6) prospectively collected data for 12 months, starting from November 2015, on perioperative anaphylaxis and identified 136 cases of grade 3 reactions [3]. The overall incidence of perioperative anaphylaxis was 1 in 10,000 anaesthetics [4]. Of all the grade 3 to 5 anaphylaxis cases reported by NAP6, Patent Blue V dye sodium salt 2.5% (Guerbet, Roissy, France) and neuromuscular blocking agents (NMBAs) were responsible for 3.4% and 25% of cases, respectively, with suxamethonium almost twice as likely to cause anaphylaxis compared with other NMBAs [4].

For IgE-mediated anaphylaxis to occur, prior sensitisation to a specific substance, or immunogenic epitopes of that substance, must have taken place during a clinically silent previous exposure [2]. One of the proposed sensitisation routes is through environmental exposure to compounds containing immunogenic tertiary or quaternary ammonium epitopes [5] which may cause cross-sensitisation to one or more NMBAs [6]. Cross-sensitivity amongst NMBAs is also a common finding during allergy testing [5].

The mechanism underlying allergy to Patent Blue V dye remains unclear but, due to its use in the food and fashion industries, environmental sensitisation to the dye or a cross-reacting epitope has been suggested [4,7]. Due to the different chemical structures of Patent Blue V dye and methylene blue, cross-sensitivity is not expected but sporadic cases have been reported [7].

We present the case of a female patient who developed delayed grade 3 allergic anaphylaxis to Patent Blue V dye during oncological breast surgery with subsequent worsening of the clinical features following administration of suxamethonium.

Patent Blue V dye is also known as E131, Acid Blue 3 and disulfine blue and is uniquely identified by Chemical Abstract Service (CAS) number 3536-49-0.

## Case presentation

A female in her 60s (ASA 2, 78 kg, BMI: 30) attended the operating theatre for an ultrasound scout-wide local excision and sentinel node biopsy of the breast. Her past medical history included hypertension and anxiety for which she was taking propranolol and citalopram. The patient had undergone three uneventful general anaesthetics: two for orthopaedic procedures within the last three years and one in the 1990s for gynaecological surgery. There were no known drug allergies.

Prior to induction of anaesthesia, the surgeon injected 2 mL of Patent Blue V dye intradermally in the subareolar region. Timings throughout this report are expressed in hours and minutes (hh:mm) from the time of dye injection which we consider to be time 00:00. Anaesthesia was induced with 140 mg of propofol and 200 mcg of fentanyl given intravenously (IV) (00:05). A supraglottic airway device (iGel® size 4, Intersurgical, Wokingham, UK) was sited and the patient ventilated with FiO2 0.6. Anaesthesia was maintained with sevoflurane, at an end-tidal concentration of 1.8%. After positioning the patient on the operating table, the surgeon infiltrated the breast with a solution of levobupivacaine 0.25% and adrenaline 1:200,000. Dexamethasone 6.6 mg, granisetron 1 mg and paracetamol 1 g were then administered IV in rapid succession during preparation of the surgical field with a topical alcohol and chlorhexidine-based solution (time 00:10). Morphine 5 mg IV was given at skin incision (time 00:20). A first episode of hypotension (68/45) was treated with ephedrine 6 mg IV (time 00:25) with a transient increase in blood pressure (112/72). A new episode of hypotension coupled with a decrease in the end-tidal CO_2_ (EtCO_2_) trace was then observed (time 00:40). This progressed to a further decrease in EtCO_2_, inability to effectively ventilate the patient both with and without the supraglottic airway device and worsening hypoxaemia, at which point emergent help was requested (time 00:43).

Suxamethonium 100 mg IV was given to facilitate endotracheal intubation (time 00:45). The first attempt at direct laryngoscopy was unsuccessful due to poor view of the larynx. The second attempt, by a more experienced anaesthetist, appeared to be successful but resulted in a flat EtCO_2_ trace and inability to ventilate hence the endotracheal tube was immediately removed. A third attempt at endotracheal intubation resulted again in a poor EtCO_2_ trace and high airway pressures despite direct visual confirmation by two senior anaesthetists that the endotracheal tube was sited through the glottis (time 00:50). A radial arterial line for invasive blood pressure monitoring and a wide bore cannula were sited. Hypotension continued to worsen to a minimum of 65/38 (time 00:50). Throughout manipulation of the airway, bag-mask ventilation with FiO_2_ 100% was difficult, resulting in hypoxaemia and arterial oxygen saturation (SaO_2_) readings as low as 65%. Auscultation of the chest revealed a bilateral wheeze which coupled with worsening hypotension suggested a working diagnosis of anaphylaxis. Two boluses of adrenaline 50 mcg IV were given (time 00:55 and 01:05) followed by a 0.1 mcg.kg.min^-1^ adrenaline infusion resulting in a good cardiovascular response and slow resolution of the bronchospasm. Resuscitation was supported by a rapid infusion of 3 litres of crystalloid. Hydrocortisone 100 mg IV was also given (time 01:30). No cardiopulmonary resuscitation (CPR) was required.

By the time the reaction occurred, excision of the tumour had already been carried out and a decision was made by the surgical team to close the wound without removing the sentinel node. The patient was resuscitated and transferred to the ICU where she made a full recovery.

Following the event, the patient and the next of kin were debriefed by the consultant anaesthetist.

The patient had to return to the operating theatre 16 hours after the initial surgery to control acute post-operative bleeding in the breast. Given the need to provide an emergency general anaesthetic to evacuate the haematoma, the inherent risk of a further allergic reaction occurring during this procedure could only be partially mitigated by avoiding some, but not all, the agents that may have precipitated the primary anaphylactic reaction. Surgical haemostasis and sentinel node biopsy were obtained uneventfully under general anaesthesia. The patient’s airway was maintained with an iGel® size 4. Propofol, fentanyl, dexamethasone, morphine, metaraminol and sevoflurane were used without sequelae during the procedure. Even though endotracheal intubation was not carried out, rocuronium and sugammadex were also administered to the patient with no adverse outcome.

Blood samples taken at the time of the reaction were tested for mast cell tryptase levels and showed a dynamic rise with a return to a normal baseline (Table 1). The first of these blood samples was also tested for some specific IgEs against common allergens which were all negative even though the total serum IgE titre was elevated (Table 1).

**Table 1:** Mast cell tryptase levels, total serum IgE and specific IgE results. Sampling times are expressed in hours:minutes from the time of injection of Patent Blue V dye. Measurements and results that are not applicable are marked as '-'.

Time from Patent Blue V dye injection (hours:minutes)	01:20	02:30	04:40	19:20
Mast cell tryptase µg.L^-1^ (normal range: 2-14 µg.L^-1^)	16.4	18	17.4	6.2
Total serum IgE kU.L^-1^ (normal range: 0-100 kU.L^-1^)	830	-	-	-
Chlorhexidine IgE kU.L^-1^ (normal range: 0-0.35 kU.AL^-1^)	0.07	-	-	-
Latex IgE kU.L^1^ (normal range: 0-0.35 kU.AL^-1^)	0.07	-	-	-

A full allergy work-up was carried out 3 months and 10 days post-event. Skin prick tests (SPT), intradermal tests (IDT) and subcutaneous challenge tests (SCT) were performed for most allergens the patient came in contact with during surgery as well as against a panel of potential cross-reacting substances (Table 2).

**Table 2:** Results from allergy clinic for skin prick tests, intra-dermal tests and subcutaneous challenge tests. Wheal sizes are reported in millimetres. SPT: skin prick tests; IDT: intra dermal tests; SCT: subcutaneous challenge tests; mm: millimetres Measurements and results that are not applicable are marked as '-'.

Substance	SPT			IDT			SCT
	Concentration (mg.ml ^-1^)	Dilution (1 in x)	Result (mm)	Concentration (µg.ml ^-1^)	Dilution (1 in x)	Result (increase in size of test in mm)	Result
Histamine (positive control)	10	1	6/6	-			-
0.9% NaCl (negative control)	9	1	0/0	-			-
Fentanyl	0.05	1	0/0	5	10	0	-
Propofol	10	1	0/0	1000	10	0	-
Suxamethonium	10	5	0/2	100	500	4x5 to 10x12 Positive	-
Atracurium	1	10	0/0	10	1000	0	-
Rocuronium	10	1	0/0	50	200	0	-
Cisatracurium	2	1	0/0	-			-
Patent Blue V dye	25	1	0/2	2500	10	5x4 to 10x8 Positive	-
Methylene blue	10	1	0/0	100	100	5x3 to 10x12 Positive	-
Granisetron	1	1	0/0	100	10	0	-
Dexamethasone	3.3	1	0/0	-			Negative
Paracetamol	10	1	0/0	1000	10	0	-
Levobupivacaine	2.5	1	0/0	-			Negative
Chlorhexidine	5	1	0/0	5	1000	0	-
Latex	0.2	1	0/0	-			-

Skin tests were positive for Patent Blue V dye, methylene blue and suxamethonium. It is not certain if a dual allergy was responsible for the biphasic nature of the reaction but sensitivity to both Patent Blue V dye and suxamethonium offers an explanation as to why the symptoms of anaphylaxis to the dye worsened after administration of the NMBA. Whilst allergy tests were suggestive of concurrent dual anaphylaxis to Patent Blue V dye and suxamethonium, this could not be proven without undertaking a challenge test with suxamethonium, which was not considered due to the risks associated and lack of clinical benefit for the patient. Regardless of the actual mechanism responsible for the reaction and based on the positive test results, the patient was counselled to avoid blue dyes and suxamethonium.

The specificity of skin testing against NMBAs and the clinical significance of sensitisation to different NMBAs remains unclear [8]. Allergy testing for all potential precipitating and cross-reacting drugs was key in reducing the prospective risk of perioperative anaphylaxis in this patient and may explain the biphasic nature of the reaction and why it is possible that the deterioration in clinical features was precipitated by a second agent rather than solely a natural progression of the initial reaction.

## Discussion

Cross-linking of antigens to specific IgE receptors on the surface of mast cells results in their degranulation which is responsible for some of the clinical features of allergic anaphylaxis. Most cases of perioperative allergic anaphylaxis occur at the time of induction of anaesthesia [2]. The latency between exposure to an allergen and the onset of symptoms may vary depending on the route by which sensitised individuals come into contact with an allergen.

NAP6 reported Patent Blue V dye as the fourth most common cause of perioperative anaphylaxis with 14.6 cases per 100,000 administrations, giving it the second highest incidence rate in the study behind teicoplanin and exceeding that of suxamethonium [4]. Most patients developed symptoms between 5 and 30 minutes of dye injection, although it can exceed 60 minutes [4,7]. Hypotension was the most likely presentation followed by hypoxaemia [9]. In our case, after dye injection, the areola was infiltrated with levobupivacaine with adrenaline, which may further delay systemic absorption of the dye from the subareolar region due to local vasoconstriction. In line with other reports [7,9-10], our patient developed hypotension insidiously between 25 and 40 minutes of dye injection, followed by a decrease in EtCO_2_. Auscultation identified widespread wheeze prompting a diagnosis of bronchospasm. Due to poor ventilation, a decision to swap the supraglottic airway device for an endotracheal tube was made and suxamethonium 100 mg IV was administered (time 00:45). Immediately after this, a rapid clinical deterioration ensued, and the patient became impossible to ventilate. Laryngeal oedema was not detected during endotracheal intubation attempts, making it an unlikely cause for the added respiratory sounds heard on auscultation and the drop in EtCO_2_. Ventilation remained challenging even after successful tracheal intubation due to worsening acute bronchospasm. This, coupled with a significant drop in blood pressure prompted a working diagnosis of perioperative anaphylaxis which we assumed to be secondary to Patent Blue V dye administration.

Patent Blue V dye is known to absorb light of a similar wavelength to that used by pulse oximeters to detect deoxyhaemoglobin resulting in falsely low readings [10]. This phenomenon tends to occur 10 to 15 minutes after intradermal injection around the areola [11]. In the context of perioperative anaphylaxis, this interference may further complicate clinical management and evaluation of resuscitative end-points such as haemoglobin saturation.

In contrast to Patent Blue V dye, bronchospasm is the most common presentation in anaphylaxis induced by suxamethonium [4]. Compared to other NMBAs, suxamethonium is almost twice as likely to cause anaphylaxis, with 11.1 cases per 100,000 administrations [4].

At the time of the events described above, we explained the worsening hypotension, acute bronchospasm and general deterioration in the clinical picture as a natural progression of the anaphylactic reaction to the dye and we did not consider this to be a second and unrelated allergic reaction to another drug given during resuscitation. Although this may be exceedingly rare and difficult to diagnose during a critical incident, our case raises it as a possibility.

Most cases of anaphylaxis to NMBAs occur in patients who are drug naïve. Sensitisation is thought to occur from exposure to compounds containing tertiary substituted ammonium groups, quaternary substituted ammonium groups, or both, such as cosmetics or disinfectants [6]. While these immunogenic epitopes are known to be the IgE recognition sites of NMBAs [5], the IgE recognition sites against vital dyes remain elusive [7].

Our patient had undergone laparoscopic surgery several years before for a gynaecological procedure without any complications. We have no information regarding this procedure, but exposure and possible sensitisation to an NMBA may have occurred at the time. This may offer an alternative explanation to environmental sensitisation, or a false positive skin test, for the positive IDT to Suxamethonium identified during follow-up.

As highlighted by the NAP6 report post-anaphylaxis allergy testing and follow-up are often suboptimal [12]. IgE anaphylaxis carries high risks during re-exposure and failure to test for all potential culprit agents and the most common cross-sensitive compounds, together with poor communication, may put patients at risk of further preventable drug reactions. For the post-anaphylaxis follow-up to be meaningful, samples obtained during the index event must be collected in a timely manner and documented scrupulously. In our case, both sampling and allergy testing were carried out rigorously and in accordance with current recommendations [13] allowing the identification of sensitivities to suxamethonium, Patent Blue V dye and Methylene blue.

While biphasic reactions to Patent Blue V dye have been reported [14] we are not aware of any other case where this particularly rare clinical presentation may have been due to two different and unrelated drugs administered in rapid succession during general anaesthesia.

## Conclusions

This case confirms the need for thorough post-anaphylaxis investigation with allergy testing covering not only administered drugs but also those known to cross-react with each other. Testing with a panel of commonly used NMBAs, alongside the reference drug, allowed us to confirm that rocuronium remained a safer NMBA for future use even after the recent administration. In addition, testing for both methylene blue and Patent Blue V dye, which are the most commonly used dyes in clinical practice, highlighted a rare cross-sensitivity. Thanks to rigorous post-anaphylaxis follow-up, the patient was given clear information on what drugs to avoid and what safer options were available for anaesthesia.

In summary, we describe an unusual case of perioperative anaphylaxis with sensitisation to two of the administered drugs and cross-sensitivity to a third drug (methylene blue), all of which were identified during allergy clinic testing.
